# Cognitive Social Capital in Community and Mental Health of the Elderly in China: The Moderating Effect of Age, Education, and Income

**DOI:** 10.3390/healthcare13070794

**Published:** 2025-04-02

**Authors:** Yaling Luo, Shaohua Zhu, Fan Yang, Yadan Li, Shuhan Yan, Yao Jiang, Jiaxi Bai

**Affiliations:** 1 Department of Labor and Social Security, School of Public Administration, Sichuan University, Chengdu 610065, China; luoyy@scu.edu.cn (Y.L.); shaohua@stu.scu.edu.cn (S.Z.); yangfan1987@scu.edu.cn (F.Y.); 2022225010090@stu.scu.edu.cn (Y.L.); 2023225010118@stu.scu.edu.cn (J.B.); 2 School of Sociology, Nankai University, Tianjin 300350, China; yanshuhan@mail.nankai.edu.cn

**Keywords:** cognitive social capital, mental health, elderly, moderating effect, China

## Abstract

**Background:** With the increasingly severe trend of population aging, the well-being of the elderly is receiving growing attention. This study aimed to investigate the association between cognitive social capital in the community (familiarity with community members, trust in community members, and sense of security in the community where they live) and the mental health among older individuals in China and to examine how age, education, and income moderate this relationship. **Methods:** To achieve this, we utilized nationally representative data (*n* = 2301) from the China Labor-Force Dynamics Survey (CLDS) 2018, and we assessed whether older adults’ mental health was associated with cognitive social capital in the community. Cognitive social capital includes familiarity with and trust in other members living in the same community together with the sense of security within the community where the older individuals reside. The marginal effect was applied to analyze how age, education, and income moderate the impact of community-based cognitive social capital on the mental health of older individuals. **Results:** Our findings indicate that the cognitive social capital within communities is significantly linked to the mental health of older adults. Additionally, age, education, and income serve as crucial moderators in this relationship. **Conclusions:** Strategies to enhance the cognitive social capital of older adults in the community are beneficial for their mental health, which deserves policymakers’ further attention.

## 1. Introduction

The mental health problem faced by older individuals has always been a major public health and social problem [1,2,3,4]. These mental health problems may have multiple causes and be expressed in multiple dimensions. Some empty-nested older parents may suffer loneliness as their children grow up and leave home [5,6,7]. Instead, if grown-up children live with their parents, the generation gap between children and parents may lead to conflicts, which may also result in mental health problems for the elderly [8,9,10]. Moreover, in the late stage of life, the elderly may also have mental health problems due to illness or fear of death [11]. Therefore, it is vital to explore the influencing factors on the mental health of older adults to improve their mental health.

By the end of 2021, the National Health Commission of China (NHCC) reported that the population aged 60 and older in China had reached 267 million, representing 18.9% of the overall population [12]. Within this demographic, individuals aged 65 and older exceeded 200 million, representing 14.2% of the overall population [12]. By 2025, it is projected that the demographic of individuals who are 60 years old and older in China will surpass 300 million, constituting more than 20% of the total population, thereby marking the onset of moderate aging [12]. Around 2035, this demographic is expected to exceed 400 million, making up over 30% of the total population and signaling a transition to severe aging [12]. Given the substantial size of this elderly population, addressing their mental health has become increasingly crucial. The concern for the well-being of the elderly not only reflects the conscience of society but also symbolizes advancements in societal development and modernization.

Mobility is hindered with age, and the incidences of cancer, dementia, and mental disorders also increase. The scope of activities of the elderly is thus limited, generally within their living community. Therefore, as a regional public good, the social capital in the community where individuals live is particularly important for the elderly, which may have an important effect on their mental health [13,14,15]. However, the exploration of this topic is not currently deep enough, and there has been no clear conclusion about the impact of social capital in the community on the mental health of older adults, especially from a cognitive social capital perspective, with depression being one of its most prevalent and impactful components. Therefore, in this study, we focus on geriatric depression as a key measure of mental health. This paper examines the effects of community-based cognitive social capital on the mental health of the elderly, with a focus on how age, education, and income moderate this relationship. To achieve this, we utilized nationally representative data from the 2018 China Labor Dynamics Survey (CLDS), analyzing a sample of 2301 respondents aged 60 to 89 years [16,17].

This study makes several contributions: First, we examined the effect of community-based social capital on elderly mental health from a cognitive perspective, considering the actual scope of activities for older adults. This advanced our theoretical understanding of the factors influencing the mental health of elderly people. Second, by employing marginal effect analysis, we investigated the moderating effects of age, education, and income on the relationship between community-based cognitive social capital and the mental health of the elderly. This gave us a nuanced view of how community-based cognitive social capital affects the mental health in older populations. The findings offer policy insights for enhancing mental health and fostering successful aging in China and other countries and regions with similar demographic and social contexts.

## 2. Theoretical Analysis

### 2.1. Definition of Social Capital

Generally, according to social capital theory, social capital encompasses the networks for relationships among individuals or groups, including social networks, reciprocal norms, and the trust developed through these interactions. These elements represent valuable resources derived from individuals’ positions within the social structure [18,19]. Social capital is a concept with multiple perspectives. Therefore, social capital has a variety of manifestations. Uphoff distinguished social capital in two forms: structural and cognitive, each characterized by internal connections [20]. Structural social capital specifically refers to the extent of individuals’ participation in their social networks, formal or informal associations, and civic activities, focusing on their actions and engagements [21,22]. Conversely, cognitive social capital refers to individuals’ perceptions of trust and reciprocity, emphasizing their emotional and attitudinal experiences [23].

A few existing empirical studies explored the influences of social capital on older adults’ self-rated physical health [23], subjective well-being [21], and psychological fragility [24]. These results indicated that individual-level cognitive social capital tends to have more substantial and significant effects on promoting older people’s evaluations of self-rated physical health, improving an older person’s subjective well-being and declining psychological fragility than structural social capital [21,23,24]. Some studies suggest that these findings may be due to older adults’ low participation in social activities, which can be attributed to their declining health and reduced mobility [21,25]. Because of the significance of community-based social capital for the health outcomes of older individuals, however, limited research has concentrated on the effect of cognitive social capital within older people’s living community on their mental health. Therefore, this study examined the relationship between community-based cognitive social capital among older adults and their mental health, and the moderating effect of older adults’ age, education attainments, and income level.

### 2.2. Cognitive Social Capital and Older People’s Mental Health

Based on social capital theory, we propose that cognitive social capital in the community among older people comprises three latent mechanisms: the elderly’s familiarity with community members, their trust in community members, and their sense of security in the community where they live. The elderly’s degree of familiarity with community members represents the older people’s perception of reciprocity among community members. Meanwhile, the degree of trust in community members and the feeling of security in the community indicate older people’s perception of trust in the setting of their living community.

First, when it comes to the elderly’s familiarity with community members, it should be noted that China is in transition from a traditional society to a modern society. In a traditional society, people live in a society of acquaintances, and they live in the same community with their close relatives and fellow villagers. However, in modern society, people in the same community may not know each other. In an acquaintance society, individuals are more likely to get material and spiritual help from their community when they need it. On the contrary, in modern society, people in a community first need to establish a connection to become familiar with each other, and then they may benefit from this familiar relationship. This familiar relationship among community members is particularly important for the elderly. When people are old and retired, their living scope may be narrowed, and most of the time this living scope is concentrated in the community. If the elderly are familiar with more people in the community, it means they have more social network contacts and social communication opportunities and frequencies. These contacts, support, and communication can be used to reduce the effect of negative life events on mental health and play a role in maintaining good mental health [25,26,27,28].

Second, as a key to cognitive social capital, trust may increase self-esteem and confidence in transaction processing ability, thus improving the level of mental health [29]. A study examining the relationship between trust in sources of COVID-19 information and mental health, during the initial stages of the pandemic in Bangladesh, found that trust in traditional media (e.g., television, radio, and newspapers) might help alleviate stress [30]. Additionally, research revealed that institutional trust partially mediated the relationship between perceived adversities and mental health during the pandemic [31]. If the elderly have a higher degree of trust in others in the community, they may be more likely to believe that they will be treated fairly in social affairs at the community level. Therefore, they are more confident in carrying out social affairs at the community level, which is conducive to their mental health and well-being.

Third, the sense of security comes from the individual’s separation from negative emotions such as fear, anxiety, or depression in possible danger or risk premonition, especially a state of psychological activity to meet current and future needs [32,33,34]. Research on occupational safety has demonstrated that the sense of job security exerts a substantial influence on mental health [35,36]. The elderly’s sense of community security comes from their daily life experience. These experiences include the public security situation in the community, such as the frequency of theft, robbery, and fighting. If the elderly suppose that the community where they live is safe, they may find relief when they are active in the community, which is conducive to maintaining good mental health.

### 2.3. Theoretical Analysis of the Moderating Effects of Age, Education, and Income

**Figure 1:**
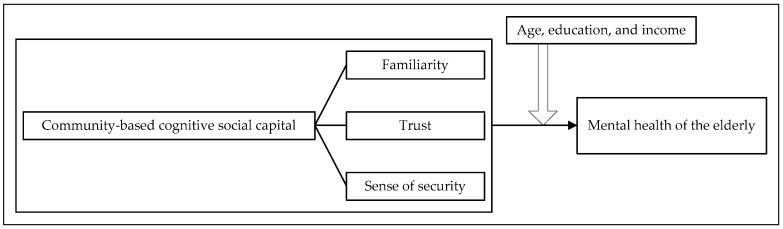
Analytical framework.

Age, education, and income may serve as moderating factors in the relationship between community-based cognitive social capital and the mental health of the elderly (Figure 1). Compared to younger elderly, older elderly are generally less physically active, have a smaller range of social activities, are less informed [37], and rely more on interactions within the community to maintain their mental health [38]. Therefore, their mental health is more influenced by familiarity with and trust in other members living in the same community. However, older elderly have more limited physical activities and less perception of community safety [39]. Their mental health is therefore less affected by community safety.

Elderly people with lower education may indicate that they have lower knowledge skills and cognitive abilities [40]. Therefore, in daily life, these older people are more inclined to seek assistance from those who have higher levels of education than them, such as seeking assistance from others for the operation of intelligent facilities and equipment [41]. If elderly people are more familiar with and trust in other community members, they are more willing to ask for help from them, and the probability of success is also higher. This is beneficial for alleviating the sense of helplessness and anxiety of these elderly people, and promotes their mental health. Similarly, the higher the education level is of elderly people, the higher are their cognitive abilities, the higher their perception of information, and the higher their perception of whether the surrounding environment is safe. Therefore, the more likely their mental health level will be affected by the sense of community environmental security.

Low-income elderly people generally lack social participation and economic opportunities [42], but their depression and stress can be eased by building good neighborhood relationships through various activities [43]. So, older people largely rely on neighborhood mutual aid. Meanwhile, low-income elderly people have low-risk preparedness for security threats, face more stress, and have poorer mental health [44]. Therefore, it can be predicted that the lower the income level of elderly people, the more their mental health level depends on familiarity with and trust in other members living in the same community and their sense of security in the community where they live.

## 3. Methods

### 3.1. Data

The data of this study were sourced from the 2018 CLDS conducted by the Center for Social Science Survey at Sun Yat-sen University. This survey is nationally representative, covering 29 provincial administrative units across China [45]. It encompasses a comprehensive range of information, including respondents’ characteristics—gender, age, education, occupation, marital status, health, economic status, and their subjective perceptions of factors like interpersonal trust and environmental security. The CLDS employs a multistage cluster and stratified probability-proportional-to-size sampling strategy to collect data. Following the removal of outliers and the handling of missing values, the final dataset comprised 2301 valid respondents aged 60 to 89 years.

### 3.2. Measures

Explained variable. The focus variable of this study was the mental health of older adults. To assess this, we utilized the Center for Epidemiological Studies Depression (CES-D) scale, which is a globally recognized tool for evaluating mental health [46]. The CES-D has been extensively validated through numerous studies, demonstrating its effectiveness in measuring mental health [45,47,48]. It consists of 20 items with total scores ranging from 20 to 80, and a lower score indicates a reduced level of depression and reflects better mental health (Cronbach’s alpha = 0.947).

Explanatory variables. The explanatory variables in this study were the elderly’s familiarity with and trust in other community members, and their sense of security in the community where they live. For each of these constructs, one item was included in the assessment. For the familiarity with other community members, respondents were asked, “How familiar are you with your neighbors and other residents in your community?”. They rated their responses on a five-point Likert scale ranging from 1 to 5, with options, namely, “very unfamiliar”, “somewhat unfamiliar”, “normal”, “somewhat familiar”, and “very familiar”.

For the trust in other community members, respondents were asked, “What degree of trust do you have with your neighbors and other residents in your community?” and rated their responses on a five-point Likert scale ranging from 1 to 5, with options, namely, “very distrustful”, “somewhat distrustful”, “normal”, “somewhat trustful”, and “very trustful”.

For the sense of security in the community where they live, respondents were asked, “How would you rate the safety of your community?” and rated their responses from 1 to 4, with options, namely, “very safe”, “somewhat safe”, “somewhat unsafe”, and “very unsafe”.

Control variables. In addition to community-based cognitive social capital, the mental health of older people is influenced by various other factors. We included control variables in the regression model analyses, encompassing gender, age, education, marital status, the logarithm of the respondent’s total annual income for 2017, smoking, drinking, and exercise. Additionally, we accounted for the regional effect based on the provinces in which the respondents resided.

### 3.3. Analysis Strategy

To estimate the effects of community-based cognitive social capital (including familiarity with and trust in other members living in the same community and their sense of security in the community where older adults live) on the mental health of older adults, we utilized ordinary least squares (OLS) regression models. The specific formulation of the OLS regression model is detailed as follows:

(1) $${Mental\;health}_{i}=\alpha_{0}+\alpha_{1}{Familiarity}_{i}+\alpha_{2}X+\varepsilon_{i}$$

(2) $${Mental\;health}_{i}=\beta_{0}+\beta_{1}{Trust}_{i}+\beta_{2}X+\delta_{i}$$

(3) $${Mental\;health}_{i}=\gamma_{0}+\gamma_{1}{Security}_{i}+\gamma_{2}X+\mu_{i}$$

where ${Mental\;health}_{i}$ denotes the explained variable reflecting the mental health of older adults, as assessed by the CES-D scale, and ${Familiarity}_{i}$, ${Trust}_{i}$, and ${Security}_{i}$ represent the explanatory variables, the elderly’s familiarity with and trust in other community members, and their sense of security in the community where older adults live, respectively. $\alpha_{0}$, $\beta_{0}$, and $\gamma_{0}$ are denoted the intercept items. $\alpha_{1}$, $\beta_{1}$, and $\gamma_{1}$ are coefficients for the explanatory variables. $\alpha_{2}$, $\beta_{2}$, and $\gamma_{2}$ are coefficients for control variables. X represents a collection of control variables, and $\varepsilon_{i}$, $\delta_{i}$, and $\mu_{i}$ are random error items.

To test the moderating effect of age, education, and income on the relationship between cognitive social capital and older adults’ mental health, we added the interactions of moderating variables and explanatory variables into the baseline OLS regression models. The specific formulation of the OLS regression model added interactions is detailed as follows:

(4) $${Mental\;health}_{i}=\alpha_{01}+\alpha_{11}{Familiarity}_{i}\times age+\alpha_{21}X+\varepsilon_{i}$$

(5) $${Mental\;health}_{i}=\alpha_{02}+\alpha_{12}{Familiarity}_{i}\times education+\alpha_{22}X+\varepsilon_{i}$$

(6) $${Mental\;health}_{i}=\alpha_{03}+\alpha_{13}{Familiarity}_{i}\times income+\alpha_{23}X+\varepsilon_{i}$$

(7) $${Mental\;health}_{i}=\beta_{01}+\beta_{11}{Trust}_{i}\times age+\beta_{21}X+\varepsilon_{i}$$

(8) $${Mental\;health}_{i}=\beta_{02}+\beta_{12}{Trust}_{i}\times education+\beta_{22}X+\varepsilon_{i}$$

(9) $${Mental\;health}_{i}=\beta_{03}+\beta_{13}{Trust}_{i}\times income+\beta_{23}X+\varepsilon_{i}$$

(10) $${Mental\;health}_{i}=\gamma_{01}+\gamma_{11}{Security}_{i}\times age+\gamma_{21}X+\varepsilon_{i}$$

(11) $${Mental\;health}_{i}=\gamma_{02}+\gamma_{12}{Security}_{i}\times education+\gamma_{22}X+\varepsilon_{i}$$

(12) $${Mental\;health}_{i}=\gamma_{03}+\gamma_{13}{Security}_{i}\times income+\gamma_{23}X+\varepsilon_{i}$$

where ${Familiarity}_{i}\times age$, ${Familiarity}_{i}\times education$, and ${Familiarity}_{i}\times income$ indicate the interactions of familiarity and age, education, and income, respectively. ${Trust}_{i}\times age$, ${Trust}_{i}\times education$, and ${Trust}_{i}\times income$ represent the interactions of trust and age, education, and income, respectively. Similarly, ${Security}_{i}\times age$, ${Security}_{i}\times education$, and ${Security}_{i}\times income$ mean the interactions of sense of security and age, education, and income, respectively.

Additionally, we used subgroup regression to test the robustness of the main empirical results. A traditional approach to analyze robustness in empirical work is to evaluate the regression model across various sub-samples. Sample splitting is a simple way to check whether a particular estimated effect is spuriously driven by a subset of the sample, such as individual characteristics [49]. Thus, in this study, based on respondents’ gender and healthy behaviors, we divided the full samples into eight subgroups to check the robustness of the main results.

## 4. Results

### 4.1. Descriptive Statistics

Table 1 indicates a descriptive analysis of all variables in this study (n = 2301). The explained variable of mental health had an average value of 27.919 (SD = 9.729). The result suggested that the mental health of the elderly in our sample was in a relatively good status. Regarding the explanatory variables, the mean value of familiarity was 4.087 (SD = 0.873). The trust had an average value of 3.916 (SD = 0.797), and the mean value of the sense of security was 1.644 (SD = 0.616).

Regarding the control variables, the average value of age was 63.892 years (SD = 4.264). The respondents’ average educational attainment was 5.743 years (SD = 4.167), which was approximately equivalent to the level of primary school. The logarithmic total annual income in 2017 on average was 9.590 (SD = 1.299). 56.5% of the sample were males, and the vast majority of respondents (90.5%) were married. For healthy behaviors, 35.2% of the elderly had a habit of smoking, 23.9% had a habit of drinking, and 28.4% had a regular exercise habit.

### 4.2. Benchmark Regression

Table 2 displays the results of three baseline regression models examining the impact of community-based cognitive social capital (encompassing older adults’ familiarity with and trust in other community members, as well as their sense of security in the community where they reside) on their mental health.

The coefficient of familiarity was significant and negative (coefficient = −0.712, *p* < 0.01), and the variable of trust (coefficient = −1.322, *p* < 0.01) showed a significant correlation with the mental health of elders. The above results revealed the more that older people are familiar with and trust in other members living in the same community, the lower are their depressive tendencies and the better their mental health. Furthermore, the variable of sense of security (coefficient = 1.123, *p* < 0.01) was significantly associated with mental health. It suggested that elderly people believed that the safer their community, the higher was the probability of their mental health level being better.

Meanwhile, gender, education, income, and the habit of exercise were significantly related to the respondents’ mental health. Specifically, the results in column (1) of Table 2 were estimated using equation (1). It can be observed that compared with women, men have better mental health (coefficient = −1.870, *p* < 0.01). The higher educational level (coefficient = −0.159, *p* < 0.01) and income (coefficient = −0.351, *p* < 0.05) indicate that the older adults have better mental health.

### 4.3. Robustness Check of Subgroup Regression

In this section, the full sample was divided into male and female groups according to gender. According to the healthy behaviors of smoking, drinking, and exercise, the full sample was categorized into six subgroups: smoking, non-smoking, drinking, non-drinking, regular exercise, and non-exercise.

The results of the eight subgroup regressions are presented in Table 3, Table 4 and Table 5. The results showed that the coefficients of the variable of familiarity with and trust in other community members, as well as the sense of security within the community were almost all significant across the different eight subgroups (Table 3, Table 4 and Table 5). Thus, these results suggested that community-based cognitive social capital has relatively stable and positive effects on the mental health of the Chinese elderly.

### 4.4. The Moderating Effect of Age, Education, and Income

**Figure 2:**
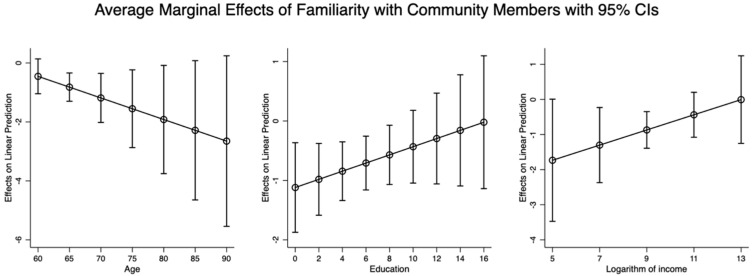
The average marginal effect of familiarity with other community members on mental health of elderly with different ages, education, and logarithm of income.

**Figure 3:**
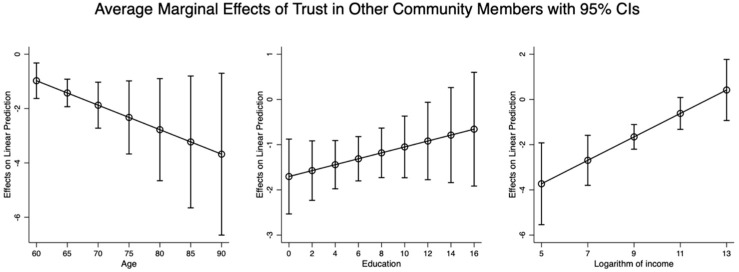
The average marginal effect of trust in other community members on mental health of elderly with different ages, education, and logarithm of income.

**Figure 4:**
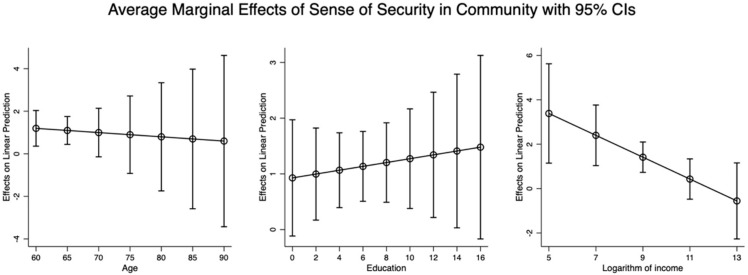
The average marginal effect of sense of security in community on mental health of elderly with different ages, education, and logarithm of income.

Figure 2, Figure 3 and Figure 4 provide the average marginal effects—familiarity with and trust in other members living in the same community, and the sense of security in the community where older adults live—on mental health among older people with different ages, education levels, and incomes. Figure 2 presents the average marginal effect of familiarity with other members living in the same community on the mental health of the elderly, increasing with increments in age and decreasing with increments in education attainment and income. The results showed that age, education, and income are important moderator mechanisms in the effect of familiarity with other members living in the same community on the mental health of the elderly. The older the elderly are, the lower their education, and the lower their income level; older people’s mental health status depends more on familiarity with residents living in the same community.

Figure 3 illustrates the average marginal effects of trust in other members living in the same community on the mental health of older adults, considering variations in age, education, and income. The results showed that the average marginal effect of trust in residents living in the same community on the mental health of the elderly strengthened with the increment in individual age, while it was undermined with education and increasing income. Collectively, age, education, and income also played moderating roles in the effects of trust in other members living in the same community on mental health.

Figure 4 provides the average marginal effects of the sense of security in the community of residence on the mental health of older adults, considering variations in age, education level, and income. The results indicated that the average marginal effect of the sense of security in the community where older adults live on the mental health of the elderly was enhanced when older adults’ education became higher. On the contrary, the average marginal effect of the sense of security in the community where older adults live weakened with age and rising income.

It can be observed from Figure 2, Figure 3 and Figure 4 that age, education, and income moderate the relationship between familiarity, trust, security, and mental health, but in different directions. As education and income increase, the effects of familiarity and trust on mental health are strengthened, whereas these effects diminish with increasing age. However, the effects of security on mental health are reduced with older age and higher income, but these effects become more pronounced as individuals’ education attainments increase.

It is important to note that the results exhibit distinct patterns due to the differing measurement scales of the key variables. Familiarity and trust are measured on a 1–5 scale, where higher values indicate greater familiarity and trust, and security is measured on a 1–4 scale, where higher values represent a lower sense of security. Mental health is measured on a 20–80 scale, where lower values indicate better mental health. Given this differing measurement, the moderating effects that appear in the opposite direction are not necessarily contradictory but instead reflect the underlying scale differences.

Overall, based on the above-mentioned results in Figure 2, Figure 3 and Figure 4, it can be concluded that older people’s age, education, and income are important moderators in the effects of familiarity with and trust in other members living in the same community and the sense of security in the community where older adults live on their mental health.

## 5. Discussion

In this paper, regarding geriatric depression as a key measure of mental health, we used nationally representative samples to explore the effect of community-based cognitive social capital on the mental health of elderly people in China. We found that cognitive social capital in community (familiarity with and trust in other members living in the same community, and the sense of security in the community where the older adults live) had a significant relationship with the mental health of elderly people. A previous study reached a similar conclusion, finding that community-based social capital significantly affects residents’ psychological health. First, familiarity with other community members positively influenced older adults’ mental health [50,51,52]. Frequent interaction with neighbors and deepening familiarity can release psychological stress [53]. Second, trust in other members living in the same community had a significant contribution to mental health in older age groups [54], and trust as a social support can directly reduce older adults’ frustration and thus avoid mental health problems [55,56]. Third, the sense of security in the community where older people live was also crucial for their mental health, according to the results of this study. To a large extent, the scope of social activities of the elderly is, most of the time, limited to the community due to their physical condition, illness, and other reasons. If a safety accident occurs in the community, such as a fight or robbery, it may affect the elderly’s sense of security towards their living community, thereby damaging their mental health. Therefore, creating a safe community environment is very necessary for the health and welfare of the elderly.

In addition, considering that an individual’s gender and lifestyle habits may affect an individual’s mental health [29], the separate group regressions were conducted by gender, smoking, drinking, and exercise, and the results were almost consistent with the results of the base regression, where community-based cognitive social capital still positively influenced older adults’ mental health. It should be noted that among the eight subgroups, the drinking and female subgroups in Table 5 showed non-significant results. However, despite the non-significance, the regression coefficients for sense of security remain positive, consistent with the patterns observed in the other subgroups. This suggests that while the statistical significance varies, the overall direction of the relationship is stable across different groups. Therefore, our findings remain robust.

Regarding the moderating effects of age, education, and income, we found that, with increasing age, the mental health of older adults becomes more dependent on familiarity with and trust in residents of the same community. The older the elderly, in daily life, the greater the probability of seeking assistance from other members in the same community. The more that elderly people are familiar with and trust in community members, the more conducive it is for them to seek help. Successfully obtaining help will be beneficial for the elderly to have a pleasant mood and good mental health. Therefore, creating more opportunities for the elderly to connect with other members of the community, increasing their familiarity with and trust in community members, is beneficial for their mental health and well-being. Community neighborhood committees and public welfare organizations can carry out social networking activities suitable for the elderly in the community, such as playing chess and cards, practicing calligraphy and painting, watching movies, reading together, and organizing sports activities suitable for the elderly. Young people in the community can act as volunteers and establish operational mechanisms similar to time banks. The funding for these events can be obtained through government funding and corporate or individual donations.

In terms of the sense of security in the community where older adults live, results, that the younger the elderly the more their mental health is dependent on the sense of security in the community they reside in, were discovered. It may be attributed to younger elderly people probably having better physical fitness than older elderly people. The younger elderly people are, the more likely they are to engage in outdoor activities, be exposed to the complex and changing environment in the community, and be more dependent on the community to provide safety in the face of outside risks. Therefore, on the premise of improving community safety hardware facilities and improving the community safety environment, it is necessary to increase community safety publicity for younger elderly people, timely understand their focus on community safety, eliminate their doubts about community safety, and enhance their sense of community safety. Once an unexpected safety accident occurs in the community, it is necessary to handle it promptly and effectively to prevent the long-term effects on the mental health of the elderly.

The results indicated that the effects of familiarity with and trust in other members of their community had a more evident influence on the mental health of older adults with lower levels of education. Older adults with limited educational attainments may generally have low knowledge skills and cognitive abilities [40]. Therefore, in daily life, these elderly people tend to seek help from other people with higher levels of education, for instance, by seeking assistance from others for the operation of intelligent facilities and equipment [41]. If elderly people are more familiar with and trust in other community members, they are more willing to ask for help from them, and the possibility of success is also higher. This is beneficial for alleviating the sense of helplessness and anxiety of these elderly people and is consequently good for their mental health.

However, we found that older adults with higher levels of education rely more on their sense of security in the community they reside in for their mental health, after controlling for other factors. Similarly, the higher the education level is of elderly people, the higher their cognitive abilities, the higher their perception of information, and the higher their perception of whether the surrounding environment is safe. Therefore, the more likely their mental health level is going to be affected by the sense of community environmental security. Thus, increasing the sense of community security of elderly people with higher levels of education is beneficial for their mental health.

Finally, the lower the income level of elderly people, the more dependent their mental health level is on familiarity with and trust in other members living in the same community, and on the sense of security in the community where older adults live. The material deprivation that may result from low-income levels is in turn more dependent on enthusiastic community neighborhoods. For this group of elderly, they have a great need to familiarize and trust others and to gain emotional support through increased social interaction with others in the community to reduce mental depression and inner loneliness [57,58]. Overall, the construction of community-based cognitive social capital for low-income elderly people is crucial for their mental health, which requires special attention from policy makers.

This study also has some limitations that deserve attention. First, due to data availability, this study used cross-sectional data instead of panel data, and the relationship between the dynamic of cognitive social capital changes and older adults’ mental health could not be observed. Therefore, future studies should be conducted using panel data to explore in greater detail. Second, although the regression process integrates factors such as income, education, age, and living habits, it is possible that health care and other factors may also be important. Therefore, follow-up studies should further consider other potential influencing factors.

## 6. Conclusions

In summary, this study innovatively examined the effects of community-based cognitive social capital on the mental health of Chinese older adults and explored the moderator effects of age, education, and income. Findings indicate that community-based cognitive social capital (familiarity with and trust in other members living in the same community, and the sense of security in the community where they live) had a significant relationship with older adults’ mental health. Age, education, and income were the vital moderator mechanisms in the relationship between community-based cognitive social capital and the mental health of older adults. Strategies to enhance the cognitive social capital of the elderly at the community level are beneficial for their mental health. This research offers valuable policy implications for promoting mental health and achieving successful aging of the elderly in China and other countries and regions with similar situations.

## Figures and Tables

**Table 1:** Variable definition and descriptive statistics.

Variable	Definition	Mean	SD	Min	Max
Explained variable					
Mental health	Total score of the CES-D scale ranges from “20” to “80”.	27.919	9.729	20	78
Explanatory variable					
Familiarity	Familiarity with other community members	4.087	0.873	1	5
Trust	Trust in other community members	3.916	0.797	1	5
Sense of security	Sense of security in community	1.644	0.616	1	4
Control variable					
Age	Years old	63.892	4.264	60	89
Education	Years of schooling education	5.743	4.167	0	16
Logarithm of income	Logarithm of total annual income of respondent in 2017.	9.590	1.299	5.704	12.676
		Frequency		Precentage	
Gender	Male	1300		56.50	
	Female	1001		43.50	
Marital status	Married	2083		90.53	
	Unmarried	218		9.47	
Smoking	Having habit of smoking	810		35.20	
	Otherwise	1491		64.80	
Drinking	Having habit of drinking	550		23.90	
	Otherwise	1751		76.10	
Exercise	Having habit of exercise	654		28.42	
	Otherwise	1647		71.58	

Notes: ‘CES-D’ means the Center for Epidemiological Studies Depression scale, and SD means the standard deviation.

**Table 2:** Influence of social capital in community (familiarity with and trust in other community members, and their sense of security in community) on the mental health of elderly.

Variable	(1)	(2)	(3)
Familiarity	−0.712 ***		
	(0.231)		
Trust		−1.322 ***	
		(0.250)	
Sense of security			1.123 ***
			(0.319)
Gender	−1.870 ***	−1.878 ***	−1.731 ***
	(0.523)	(0.521)	(0.524)
Age	−0.081 *	−0.078	−0.087 *
	(0.048)	(0.048)	(0.048)
Education	−0.159 ***	−0.159 ***	−0.159 ***
	(0.054)	(0.054)	(0.054)
Marital status	−0.811	−0.798	−0.843
	(0.671)	(0.668)	(0.670)
Logarithm of income	−0.351 **	−0.353 **	−0.332 **
	(0.169)	(0.168)	(0.168)
Smoking	0.074	0.136	−0.013
	(0.511)	(0.509)	(0.510)
Drinking	−0.396	−0.441	−0.465
	(0.520)	(0.518)	(0.520)
Exercise	−1.346 ***	−1.329 ***	−1.321 ***
	(0.456)	(0.455)	(0.456)
Regional effect	Yes	Yes	Yes
*n*	2301	2301	2301
R^2^	0.112	0.120	0.114

Notes: Robust standard errors in parentheses; *** *p* < 0.01, ** *p* < 0.05, * *p* < 0.1; ‘Yes’ means the variable is added to the model.

**Table 3:** Influence of familiarity with other community members on the mental health of elderly in different groups.

Variable	(1) Male	(2) Female	(3) Smoking	(4) No Smoking	(5) Drinking	(6) No Drinking	(7) Exercise	(8) No Exercise
Familiarity	−0.684 **	−0.709 *	−0.907 **	−0.640 **	−1.205 ***	−0.557 **	−0.862 **	−0.688 **
	(0.280)	(0.392)	(0.377)	(0.294)	(0.441)	(0.272)	(0.379)	(0.288)
Gender			−2.987 **	−2.095 ***	−3.946 ***	−1.837 ***	−2.105 **	−2.046 ***
			(1.512)	(0.599)	(1.398)	(0.596)	(0.919)	(0.639)
Age	−0.134 **	−0.018	−0.254 ***	−0.002	−0.088	−0.074	−0.099	−0.073
	(0.056)	(0.088)	(0.081)	(0.061)	(0.088)	(0.058)	(0.081)	(0.060)
Education	−0.117 *	−0.213 **	−0.128	−0.160 **	−0.037	−0.178 ***	−0.204 **	−0.136 **
	(0.068)	(0.090)	(0.093)	(0.068)	(0.105)	(0.064)	(0.088)	(0.068)
Marital status	−0.229	−1.140	−1.914 *	−0.084	−2.574 **	−0.009	0.510	−1.199
	(0.876)	(1.055)	(1.090)	(0.859)	(1.121)	(0.825)	(1.226)	(0.804)
Logarithm of income	−0.743 ***	0.045	−0.778 ***	−0.166	−0.938 ***	−0.155	−0.496	−0.340 *
	(0.213)	(0.274)	(0.280)	(0.214)	(0.307)	(0.201)	(0.317)	(0.200)
Smoking	0.025	0.706			−0.555	0.423	−0.089	0.315
	(0.507)	(1.683)			(0.803)	(0.644)	(0.869)	(0.628)
Drinking	−0.867 *	1.673	−1.425 **	0.618			0.468	−0.725
	(0.519)	(1.661)	(0.658)	(0.841)			(0.855)	(0.647)
Exercise	−1.141 **	−1.366 *	−1.466 **	−1.299 **	−0.724	−1.529 ***		
	(0.551)	(0.789)	(0.730)	(0.593)	(0.818)	(0.545)		
Regional effect	Yes	Yes	Yes	Yes	Yes	Yes	Yes	Yes
*n*	1300	1001	810	1491	550	1751	654	1647
R^2^	0.106	0.123	0.142	0.111	0.204	0.101	0.178	0.099

Notes: Robust standard errors in parentheses; *** *p* < 0.01, ** *p* < 0.05, * *p* < 0.1; ‘Yes’ means the variable is added to the model.

**Table 4:** Influence of trust in other community members on the mental health of elderly in different groups.

Variable	(1) Male	(2) Female	(3) Smoking	(4) No Smoking	(5) Drinking	(6) No Drinking	(7) Exercise	(8) No Exercise
Trust	−1.269 ***	−1.323 ***	−1.706 ***	−1.156 ***	−1.945 ***	−1.110 ***	−1.310 ***	−1.332 ***
	(0.301)	(0.431)	(0.407)	(0.319)	(0.444)	(0.299)	(0.419)	(0.311)
Gender			−2.976 **	−2.122 ***	−3.779 ***	−1.866 ***	−1.979 **	−2.091 ***
			(1.500)	(0.598)	(1.382)	(0.595)	(0.917)	(0.637)
Age	−0.133 **	−0.015	−0.250 ***	−0.000	−0.078	−0.074	−0.090	−0.076
	(0.056)	(0.088)	(0.080)	(0.061)	(0.087)	(0.057)	(0.081)	(0.059)
Education	−0.118 *	−0.213 **	−0.124	−0.161 **	−0.037	−0.179 ***	−0.207 **	−0.135 **
	(0.068)	(0.089)	(0.092)	(0.068)	(0.104)	(0.063)	(0.087)	(0.068)
Marital status	−0.268	−1.095	−1.981 *	−0.050	−2.634 **	0.007	0.725	−1.246
	(0.872)	(1.052)	(1.082)	(0.857)	(1.108)	(0.823)	(1.224)	(0.801)
Logarithm of income	−0.745 ***	0.039	−0.719 ***	−0.188	−0.954 ***	−0.157	−0.496	−0.338 *
	(0.212)	(0.272)	(0.278)	(0.213)	(0.303)	(0.200)	(0.316)	(0.199)
Smoking	0.082	0.746			−0.606	0.506	−0.087	0.384
	(0.504)	(1.677)			(0.792)	(0.643)	(0.866)	(0.626)
Drinking	−0.916 *	1.524	−1.566 **	0.632			0.321	−0.743
	(0.516)	(1.656)	(0.653)	(0.839)			(0.853)	(0.644)
Exercise	−1.083 **	−1.424 *	−1.463 **	−1.286 **	−0.709	−1.509 ***		
	(0.549)	(0.787)	(0.725)	(0.591)	(0.809)	(0.544)		
Regional effect	Yes	Yes	Yes	Yes	Yes	Yes	Yes	Yes
*n*	1300	1001	810	1491	550	1751	654	1647
R^2^	0.114	0.128	0.155	0.116	0.221	0.106	0.184	0.106

Notes: Robust standard errors in parentheses; *** *p* < 0.01, ** *p* < 0.05, * *p* < 0.1; ‘Yes’ means the variable is added to the model.

**Table 5:** Influence of sense of security in the community where older adults live on the mental health of elderly in different groups.

Variable	(1) Male	(2) Female	(3) Smoking	(4) No Smoking	(5) Drinking	(6) No Drinking	(7) Exercise	(8) No Exercise
Sense of security	1.640 ***	0.527	1.936 ***	0.721 *	0.704	1.251 ***	1.415 **	1.148 ***
	(0.395)	(0.523)	(0.503)	(0.413)	(0.573)	(0.381)	(0.549)	(0.392)
Gender			−2.556 *	−2.026 ***	−3.674 ***	−1.731 ***	−2.006 **	−1.899 ***
			(1.505)	(0.600)	(1.409)	(0.596)	(0.919)	(0.639)
Age	−0.140 **	−0.024	−0.264 ***	−0.007	−0.101	−0.078	−0.109	−0.078
	(0.056)	(0.088)	(0.080)	(0.061)	(0.088)	(0.057)	(0.081)	(0.059)
Education	−0.119 *	−0.206 **	−0.137	−0.156 **	−0.061	−0.176 ***	−0.194 **	−0.141 **
	(0.068)	(0.089)	(0.092)	(0.068)	(0.105)	(0.063)	(0.087)	(0.068)
Marital status	−0.363	−1.129	−2.166 **	−0.070	−2.641 **	−0.023	0.581	−1.249
	(0.872)	(1.057)	(1.085)	(0.860)	(1.127)	(0.824)	(1.226)	(0.803)
Logarithm of income	−0.735 ***	0.082	−0.733 ***	−0.146	−0.902 ***	−0.142	−0.480	−0.323
	(0.212)	(0.273)	(0.278)	(0.214)	(0.308)	(0.200)	(0.316)	(0.200)
Smoking	−0.059	0.606			−0.767	0.374	−0.275	0.255
	(0.504)	(1.685)			(0.805)	(0.643)	(0.869)	(0.627)
Drinking	−0.951 *	1.606	−1.577 **	0.606			0.484	−0.814
	(0.516)	(1.663)	(0.654)	(0.842)			(0.854)	(0.646)
Exercise	−1.103 **	−1.353 *	−1.454 **	−1.275 **	−0.629	−1.524 ***		
	(0.549)	(0.791)	(0.726)	(0.593)	(0.823)	(0.544)		
Regional effect	Yes	Yes	Yes	Yes	Yes	Yes	Yes	Yes
*n*	1300	1001	810	1491	550	1751	654	1647
R^2^	0.113	0.121	0.152	0.110	0.195	0.104	0.180	0.100

Notes: Robust standard errors in parentheses; *** *p* < 0.01, ** *p* < 0.05, * *p* < 0.1; ‘Yes’ means the variable is added to the model.

## Data Availability

The data that support the findings of this study are available from the corresponding author upon reasonable request.

## References

[1] Alfaro A.J., Carlson C., Segal D.L., Gould C.E. (2022). Distinctions between depression and anxiety with fear of being a burden in late life. Aging Ment. Health.

[2] Domènech-Abella J., Switsers L., Mundó J., Dierckx E., Dury S., Donder L.D. (2021). The association between perceived social and physical environment and mental health among older adults: Mediating effects of loneliness. Aging Ment. Health.

[3] Hodgetts J., McLaren S., Bice B., Trezise A. (2021). The relationships between self-compassion, rumination, and depressive symptoms among older adults: The moderating role of gender. Aging Ment. Health.

[4] Simons M., Lataster J., Reijnders J., Peeters S., Janssens M., Jacobs N. (2020). Bonding personal social capital as an ingredient for positive aging and mental well-being. A study among a sample of Dutch elderly. Aging Ment. Health.

[5] Cheng P., Jin Y., Sun H., Tang Z., Zhang C., Chen Y., Zhang Q., Zhang Q., Huang F. (2015). Disparities in prevalence and risk indicators of loneliness between rural empty nest and non-empty nest older adults in Chizhou, China. Geriatr. Gerontol. Int..

[6] Mahdiyar F., Khayyer M., Hosseini S.M. (2015). Comparison between empty nest syndrome in parents, before and after their child(ren) left home. Knowl. Res. Appl. Psychol..

[7] Liu L.J., Guo Q. (2007). Loneliness and health-related quality of life for the empty nest elderly in the rural area of a mountainous county in China. Qual Life Res..

[8] Zhang X., Silverstein M. (2021). Intergenerational ambivalence, loneliness, and well-being among older adults in the United States. Innov. Aging.

[9] Takagi E., Saito Y. (2020). Japanese older adults’ loneliness, family relationships, and mortality: Does one’s living arrangement make a difference?. Geriatr. Gerontol. Int..

[10] Nicolaisen M., Thorsen K. (2014). Loneliness among men and women—A five-year follow-up study. Aging Ment. Health.

[11] Tran T., Hammarberg K., Ryan J., Lowthian J., Freak-Poli R., Owen A., Kirkman M., Curtis A., Rowe H., Brown H. (2019). Mental health trajectories among women in Australia as they age. Aging Ment. Health.

[12] National Health Commission of China (NHCC) (2022). China Will Enter the Stage of Severe Aging Around 2035. https://baijiahao.baidu.com/s?id=1744466037246712838&wfr=spider&for=pc.

[13] Forsman A.K., Herberts C., Wahlbeck K., Schierenbeck I. (2013). Understanding the role of social capital for mental wellbeing among older adults. Ageing Soc..

[14] An S., Jang Y.R. (2018). The role of social capital in the relationship between physical constraint and mental distress in older adults: A latent interaction model. Aging Ment. Health.

[15] Cramm J.M., van Dijk H.M., Nieboer A.P. (2013). The importance of neighborhood social cohesion and social capital for the well being of older adults in the community. Gerontologist.

[16] The State Council The People’s Republic of China (2018). Law on the Protection of the Rights and Interests of the Elderly of the People’s Republic of China (中华人民共和国老年人权益保障法). https://www.gov.cn/guoqing/2021-10/29/content_5647622.htm.

[17] The United Nations (UN) (2020). Policy Brief: The Impact of COVID-19 on Older Adults. https://www.un.org/sites/un2.un.org/files/2020/10/old_persons_chinese.pdf.

[18] Rodgers J., Valuev A.V., Subramanian S.V. (2019). Social capital and physical health: An updated review of the literature for 2007–2018. Soc. Sci. Med..

[19] Ehsan A.M., De Silva M.J. (2015). Social capital and common mental disorder: A systematic review. J. Epidemiol. Community Health.

[20] Uphoff N., Dasgupta P., Serageldin I. (2000). Understanding social capital: Learning from the analysis and experience of participation. Social Capital: A Multifaceted Perspective.

[21] Zhang J.Y., Tian Y.P., Lu N. (2022). Examination of the moderating role of household income in the association between cognitive social capital and subjective well-being among rural older adults in Northeast China. Res. Aging.

[22] Han Y., Chung R.Y.N. (2023). Pre-COVID-19 cognitive social capital and peri-COVID-19 depression: A prospective cohort study on the contextual moderating effect of the COVID-19 pandemic in China, 2016–2020. Health Place.

[23] Lu N., Zhang J.Y. (2019). Social capital and self-rated health among older adults living in urban China: A mediation model. Sustainability.

[24] Hu S., Jin C.H., Li S.J. (2022). Association between social capital and frailty and the mediating effect of health-promoting lifestyles in Chinese older adults: A cross-sectional study. BMC Geriatr..

[25] Sun X., Rehnberg C., Meng Q. (2009). How are individual-level social capital and poverty associated with health equity? A study from two Chinese cities. Int. J. Equity Health.

[26] Bone J.K., Bu F., Fluharty M.E., Paul E., Sonke J.K., Fancourt D. (2022). Engagement in leisure activities and depression in older adults in the United States: Longitudinal evidence from the Health and Retirement Study. Soc. Sci. Med..

[27] Sepúlveda-Loyola W., Rodríguez-Sánchez I., Pérez-Rodríguez P., Ganz F., Torralba R., Oliveira D.V., Rodríguez-Mañas L. (2020). Impact of social isolation due to COVID-19 on health in older people: Mental and physical effects and recommendations. J. Nutr. Health Aging.

[28] Luo Y., Hawkley L.C., Waite L.J., Cacioppo J.T. (2012). Loneliness, health, and mortality in old age: A national longitudinal study. Soc. Sci. Med..

[29] Dai X., Gu N. (2022). The impact of social capital on mental health: Evidence from the China Family Panel Survey. Int. J. Environ. Res. Public Health.

[30] Patwary M.M., Bardhan M., Browning M.H.E.M., Disha A.S., Haque M.Z., Billah S.M., Kabir M.P., Hossain M.R., Alam M.A., Shuvo F.K. (2022). Association between perceived trusted of COVID-19 information sources and mental health during the early stage of the pandemic in Bangladesh. Healthcare.

[31] Lee S. (2022). Subjective well-being and mental health during the pandemic outbreak: Exploring the role of institutional trust. Res. Aging.

[32] Liu N., Li X., Ding X., Liu H., Zhang X. (2023). Mediating roles of perceived social support and sense of security in the relationship between negative life events and life satisfaction among left-behind children: A cross-sectional study. Front. Psychol..

[33] Feng L., Zhong H. (2021). Interrelationships and methods for improving university students’ sense of gain, sense of security, and happiness. Front. Psychol..

[34] Wikström B. (2007). Congregate housing for old people: The importance of the physical environment. Aust. J. Prim. Health.

[35] Antino M., Ruiz-Zorrilla P., Sanz-Vergel A.I., Leon-Perez J.M., Rodriguez-Muñoz A. (2022). The role of job insecurity and work-family conflict on mental health evolution during COVID-19 lockdown. Eur. J. Work Organ. Psychol..

[36] Sheng Z., Griffin M.A. (2023). Job insecurity, employability, and mental health in the new era: A test of plausible influence mechanisms and temporal effects. Stress Health.

[37] Xu X., Zhao L. (2023). Social capital and the realization of mutual assistance for the elderly in rural areas—Based on the intermediary role of psychological capital. Int. J. Environ. Res. Public Health.

[38] Huang Z., Long C., Yi C. (2023). The relationship between neighborhood social capital and the health of Chinese urban elderly: An analysis based on CHARLS2018 data. Healthcare.

[39] Xu Z., Zhang W., Zhang X., Wang Y., Chen Q., Gao B., Li N. (2022). Multi-level social capital and subjective wellbeing among the elderly: Understanding the effect of family, workplace, community, and society social capital. Front. Public Health.

[40] Halman L.C.J.M., Luijkx R. (2006). Social capital in contemporary Europe: Evidence from the European Social Survey. Port. J. Soc. Sci..

[41] Mcnamara T.K., Gonzales E. (2011). Volunteer transitions among older adults: The role of human, social, and cultural capital in later life. J. Gerontol. Ser. B Gerontol. Soc. Am..

[42] Lee H.J., Lee D.K., Song W. (2019). Relationships between social capital, social capital satisfaction, self-esteem, and depression among elderly urban residents: Analysis of secondary survey data. Int. J. Environ. Res. Public Health.

[43] Zhu J., Liang C., Lucas J., Cheng W., Zhao Z. (2020). The influence of income and social capital on the subjective well-being of elderly Chinese people, based on a panel survey. Sustainability.

[44] Choi Y.J., Matz-Costa C. (2018). Perceived neighborhood safety, social cohesion, and psychological health of older adults. Gerontologist.

[45] Jiang Y., Xiao H., Yang F. (2023). Accompanying your children: Living without parents at different stages of pre-adulthood and individual physical and mental health in adulthood. Front. Public Health.

[46] Radloff L.S. (1977). The CES-D scale: A self report depression scale for research in the general population. Appl. Psychol. Meas..

[47] Ohrnberger J., Anselmi L., Fichera E., Sutton M. (2020). The effect of cash transfers on mental health: Opening the black box–a study from south Africa. Soc. Sci. Med..

[48] Somefun O.D., Fotso A.S. (2020). The effect of family and neighbourhood social capital on youth mental health in south Africa. J. Adolesc..

[49] Acemoglu D., Johnson S., Robinson J.A. (2001). The colonial origins of comparative development: An empirical investigation. Am. Econ. Rev..

[50] Choi E., Han K.M., Chang J., Lee Y.J., Choi K.W., Han C., Ham B.J. (2021). Social participation and depressive symptoms in community-dwelling older adults: Emotional social support as a mediator. J. Psychiatr. Res..

[51] Kemppainen J., Timonen M. (2024). Social capital and depressive symptoms: A systematic review. J. Theor. Soc. Psychol..

[52] Zhang J., Lu N. (2019). What matters most for community social capital among older adults living in urban China: The role of health and family social capital. Int. J. Environ. Res. Public Health.

[53] Laurence J., Kim H.H. (2021). Individual and community social capital, mobility restrictions, and psychological distress during the COVID-19 pandemic: A multilevel analysis of a representative US survey. Soc. Sci. Med..

[54] Nieminen T., Martelin T., Koskinen S., Aro H., Alanen E., Hyyppä M.T. (2010). Social capital as a determinant of self-rated health and psychological well-being. Int. J. Public Health.

[55] Ricciardi E., Spano G., Tinella L., Lopez A., Clemente C., Bosco A., Caffò A.O. (2023). Perceived social support mediates the relationship between use of greenspace and geriatric depression: A cross-sectional study in a sample of south-Italian older adults. Int. J. Environ. Res. Public Health.

[56] Cao W., Li L., Zhou X., Zhou C. (2015). Social capital and depression: Evidence from urban elderly in China. Aging Ment. Health.

[57] Folland S. (2008). An economic model of social capital and health. Health Econ. Policy Law.

[58] Taube E., Kristensson J., Sandberg M., Midlöv P., Jakobsson U. (2015). Loneliness and health care consumption among older people. Scand. J. Caring Sci..

